# Supplementary material for: High VEGFA Expression Is Associated with Improved Progression-Free Survival after Bevacizumab Treatment in Recurrent Glioblastoma
Source: Cancers (Basel). 2023 Apr 7;15(8):2196. doi: 10.3390/cancers15082196 (PMC10136662; doi:10.3390/cancers15082196)
Supplement: Supplementary file 1 [file cancers-15-02196-s001.zip › cancers-2264877-supplementary.pdf]

cancers

MDPI

*Supplementary materials*

# High VEGFA Expression is Associated with Improved Progression-Free Survival after Bevacizumab Treatment in Recurrent Glioblastoma

**Bárbara Alves [1,2,3], Joana Peixoto [1,2], Sofia Macedo [1,2], Jorge Pinheiro [4], Bruno Carvalho [5,6], Paula Soares [1,2,7], Jorge Lima [1,2,7], Raquel T. Lima [1,2,7]***

[1] i3S – Instituto de Investigação e Inovação em Saúde, 4200 Porto, Portugal
[2] Cancer Signalling & Metabolism Group, IPATIMUP – Institute of Molecular Pathology and Immunology of the University of Porto, 4200 Porto, Portugal
[3] School of Allied Health Sciences, Polytechnic Institute of Porto, 4200 Porto, Portugal;
[4] Department of Pathology, Centro Hospitalar Universitário S. João, 4200 Porto, Portugal;
[5] Department of Neurosurgery, Centro Hospitalar Universitário S. João, 4200 Porto, Portugal;
[6] FMUP – Faculty of Medicine of the University of Porto, 4200 Porto, Portugal
[7] Department of Pathology, FMUP – Faculty of Medicine of the University of Porto, 4200 Porto, Portugal
* Corresponding author: rlima@ipatimup.pt (R.T.L.); Tel.: +351 220 408 800

**Table S1.** Association between the proteins evaluated and the chosen IHC score (IHS) threshold values.

| **Protein** | | **YKL40** | | | **VEGFA** | | | **MMP-9** | | |
|---|---|---|---|---|---|---|---|---|---|---|
| | | **IHS ≤ 4** | **IHS > 4** | ***p* value** | **IHS ≤ 4** | **IHS > 4** | ***p* value** | **IHS = 0** | **IHS ≥ 1** | ***p* value** |
| **MMP-2** | **IHS ≤ 12** | 16 | 8 | 0.648 | 20 | 8 | 0.078 | 23 | 4 | **<0.001** |
| | **IHS = 12** | 11 | 16 | | 18 | 13 | | 25 | 5 | |
| **MMP-9** | **IHS = 0** | 28 | 18 | **<0.001** | 30 | 18 | **0.043** | | | |
| | **IHS ≥ 1** | 1 | 7 | | 7 | 2 | | | | |
| **VEGFA** | **IHS ≤ 4** | 14 | 16 | 0.710 | | | | | | |
| | **IHS > 4** | 13 | 8 | | | | | | | |

**Table S2.** Association of clinicopathological features of GB patients with the expression of MMP-2, MMP-9, VEGFA, and YKL40 according to their IHS threshold.

| Clinical Features | MMP-2 | | | MMP-9 | | | VEGFA | | | YKL40 | | |
|---|---|---|---|---|---|---|---|---|---|---|---|---|
| | IHS < 12 | IHS = 12 | *p* value | IHS = 0 | IHS ≥ 1 | *p* value | IHS ≤ 4 | IHS > 4 | *p* value | IHS ≤ 4 | IHS > 4 | *p* value |
| **Sex** | *n* = 59 | | | *n* = 61 | | | *n* = 59 | | | *n* = 55 | | |
| Masculine | 15 (53.6%) | 18 (58.1%) | 0.796 | 28 (53.8%) | 4 (44.4%) | 0.724 | 18 (47.4%) | 15 (71.4%) | 0.102 | 15 (51.7%) | 16 (61.5%) | 0.588 |
| Feminine | 13 (46.4%) | 13 (41.9%) | | 24 (46.2%) | 5 (55.6%) | | 20 (52.6%) | 6 (28.6%) | | 14 (48.3%) | 10 (38.5%) | |
| **Age** | *n* = 59 | | | *n* = 61 | | | *n* = 59 | | | *n* = 55 | | |
| < 65 | 13 (46.4%) | 15 (47.5%) | 1.000 | 27 (51.9%) | 3 (33.3%) | 0.473 | 17 (44.7%) | 11 (52.4%) | 0.598 | 15 (51.7%) | 12 (46.2%) | 0.789 |
| ≥ 65 | 15 (53.6%) | 16 (51.6%) | | 25 (48.1%) | 6 (66.7%) | | 21 (55.3%) | 10 (47.6%) | | 14 (48.3%) | 14 (53.8%) | |
| **KPS** | *n* = 58 | | | *n* = 59 | | | *n* = 58 | | | *n* = 53 | | |
| < 70 | 0 (0.0%) | 1 (3.2%) | 1.000 | 1 (2.0%) | 0 (0.0%) | 1.000 | 0 (0.0%) | 1 (4.8%) | 0.362 | 0 (0.0%) | 1 (4.0%) | 0.472 |
| ≥ 70 | 27 (100.0%) | 30 (96.8%) | | 49 (98.0%) | 9 (100.0%) | | 37 (100.0%) | 20 (95.2%) | | 28 (100.0%) | 24 (96.0%) | |
| **ECOG** | *n* = 58 | | | *n* = 61 | | | *n* = 58 | | | *n* = 53 | | |
| 0 or 1 | 26 (96.3%) | 26 (83.9%) | 0.201 | 45 (90.0%) | 8 (88.9%) | 1.000 | 34 (91.9%) | 18 (85.7%) | 0.657 | 26 (92.9%) | 23 (92.0%) | 1.000 |
| ≥ 2 | 1 (3.7%) | 5 (16.1%) | | 5 (10.0%) | 1 (11.1%) | | 3 (8.1%) | 3 (14.3%) | | 2 (7.1%) | 2 (8.0%) | |
| **MGMT Status** | *n* = 37 | | | *n* = 40 | | | *n* = 37 | | | *n* = 35 | | |
| Methylated | 7 (38.9%) | 12 (63.2%) | 0.194 | 16 (48.5%) | 4 (57.1%) | 1.000 | 12 (52.2%) | 7 (50.0%) | 1.000 | 10 (50.0%) | 8 (53.3%) | 1.000 |
| Non-methylated | 11 (61.1%) | 7 (36.8%) | | 17 (51.5%) | 3 (42.9%) | | 11 (47.8%) | 7 (50.0%) | | 10 (50.0%) | 7 (46.7%) | |
| **Tumor Laterality** | *n* = 58 | | | *n* = 60 | | | *n* = 58 | | | *n* = 54 | | |
| Unilateral | 27 (96.4%) | 29 (96.7%) | 1.000 | 49 (96.1%) | 9 (100.0%) | 1.000 | 37 (97.4%) | 19 (95.0%) | 1.000 | 27 (96.4%) | 25 (96.2%) | 1.000 |
| Bilateral | 1 (3.6%) | 1 (3.3%) | | 2 (3.9%) | 0 (0.0%) | | 1 (2.6%) | 1 (5.0%) | | 1 (3.6%) | 1 (3.8%) | |
| **Tumor Focality** | *n* = 56 | | | *n* = 58 | | | *n* = 56 | | | *n* = 52 | | |
| Unifocal | 24 (92.3%) | 27 (90.0%) | 1.000 | 45 (90.0%) | 8 (100.0%) | 1.000 | 33 (91.7%) | 18 (90.0%) | 1.000 | 26 (93.6%) | 21 (84.0%) | 0.183 |
| Multifocal or Multicentric | 2 (7.7%) | 3 (10.0%) | | 5 (10.0%) | 0 (0.0%) | | 3 (8.3%) | 2 (10.0%) | | 1 (3.7%) | 4 (16.0%) | |

**Table S2 (cont.)**

| | | | | | | | | | | | | | |
|---|---|---|---|---|---|---|---|---|---|---|---|---|---|
| **Tumor Location** | ***n* = 59** | | | ***n* = 61** | | | ***n* = 59** | | | ***n* = 57** | | | |
| Frontal | 5 (17.9%) | 7 (22.6%) | 0.920 | 9 (17.3%) | 3 (33.3%) | 0.749 | 8 (21.1%) | 4 (19.0%) | 0.350 | 5 (17.2%) | 5 (19.2%) | **0.007** |
| Temporal | 10 (37.7%) | 9 (29.0%) | | 16 (30.8%) | 3 (33.3%) | | 9 (23.7%) | 10 (47.6%) | | 8 (27.6%) | 10 (38.5%) | |
| Parietal | 5 (17.9%) | 4 (12.9%) | | 8 (15.4%) | 1 (11.1%) | | 6 (15.8%) | 3 (14.3%) | | 1(3.4%) | 8 (30.8%) | |
| Multiple | 7 (25.0%) | 9 (29.0%) | | 15 (28.8%) | 2 (22.2%) | | 13 (34.2%) | 3 (14.3%) | | 12 (41.4%) | 3 (11.5%) | |
| Others | 1 (3.6%) | 2 (6.5%) | | 4 (7.7%) | 0 (0.0%) | | 2 (5.3%) | 1 (4.8%) | | 3 (10.3%) | 0 (0.0%) | |
| **Type of Surgery** | ***n* = 54** | | | ***n* = 55** | | | ***n* = 54** | | | ***n* = 49** | | | |
| Biopsy | 1 (4.0%) | 3 (10.3%) | 0.650 | 4 (8.5%) | 0 (0.0%) | 0.177 | 3 (8.8%) | 1 (5.0 | 0.872 | 2 (7.7%) | 1 (4.3%) | 0.592 |
| Partial or subtotal resection | 12 (48.0%) | 12 (41.4%) | | 24 (51.1%) | 2 (25.0%) | | 15 (44.1%) | 9 (45.0%) | | 13 (50.0%) | 9 (39.1%) | |
| Total resection | 12 (48.0%) | 14 (48.3%) | | 19 (40.4%) | 6 (75.0%) | | 16 (47.1%) | 10 (50.0%) | | 11 (42.3%) | 13 (56.5%) | |
| **Number of Temozolomide Cycles** | ***n* = 59** | | | ***n* = 61** | | | ***n* = 59** | | | ***n* = 55** | | | |
| Median (range) | 7 (0 – 22) | 5 (0 - 42) | 0.318 | 6 (0 – 42) | 6 (2 - 18) | 0.862 | 6 (0 – 42) | 6 (0 - 21) | 0.252 | 6 (0 – 22) | 6 (0 - 42) | 0.636 |
| **Number of Bevacizumab Doses** | ***n* = 59** | | | ***n* = 61** | | | ***n* = 59** | | | ***n* = 55** | | | |
| Median (range) | 7.5 (1 – 29) | 9 (2 – 41) | 0.326 | 8 (1 – 41) | 9 (2 – 22) | 0.721 | 7 (1 – 41) | 10 (1 – 37) | 0.122 | 6 (1 – 37) | 9.5 (2 – 41) | **0.035** |

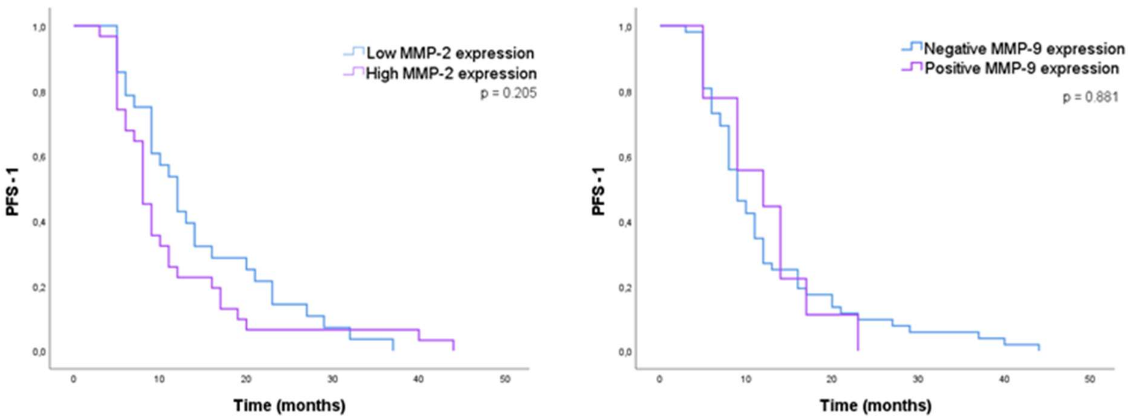


(a) (b)

**Supplementary Figure S1.** Kaplan-Meier analysis of progression-free survival (in months) after temozolomide treatment (PFS-1) according to the expression of MMP-2 **(a)**, and MMP-9 **(b)**.

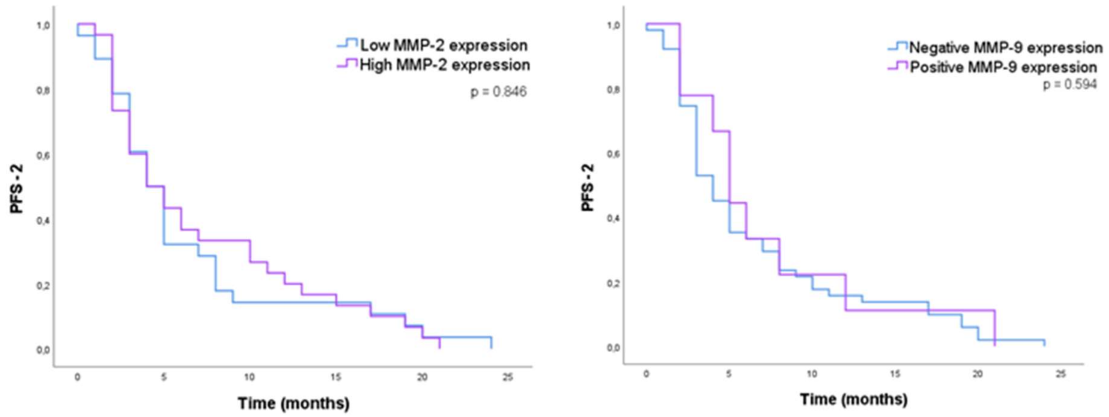


(a) (b)

**Supplementary Figure S2.** Kaplan-Meier analysis of progression-free survival (in months) after bevacizumab treatment (PFS-2) according to the expression of MMP-2 **(a)**, and MMP-9 **(b)**.

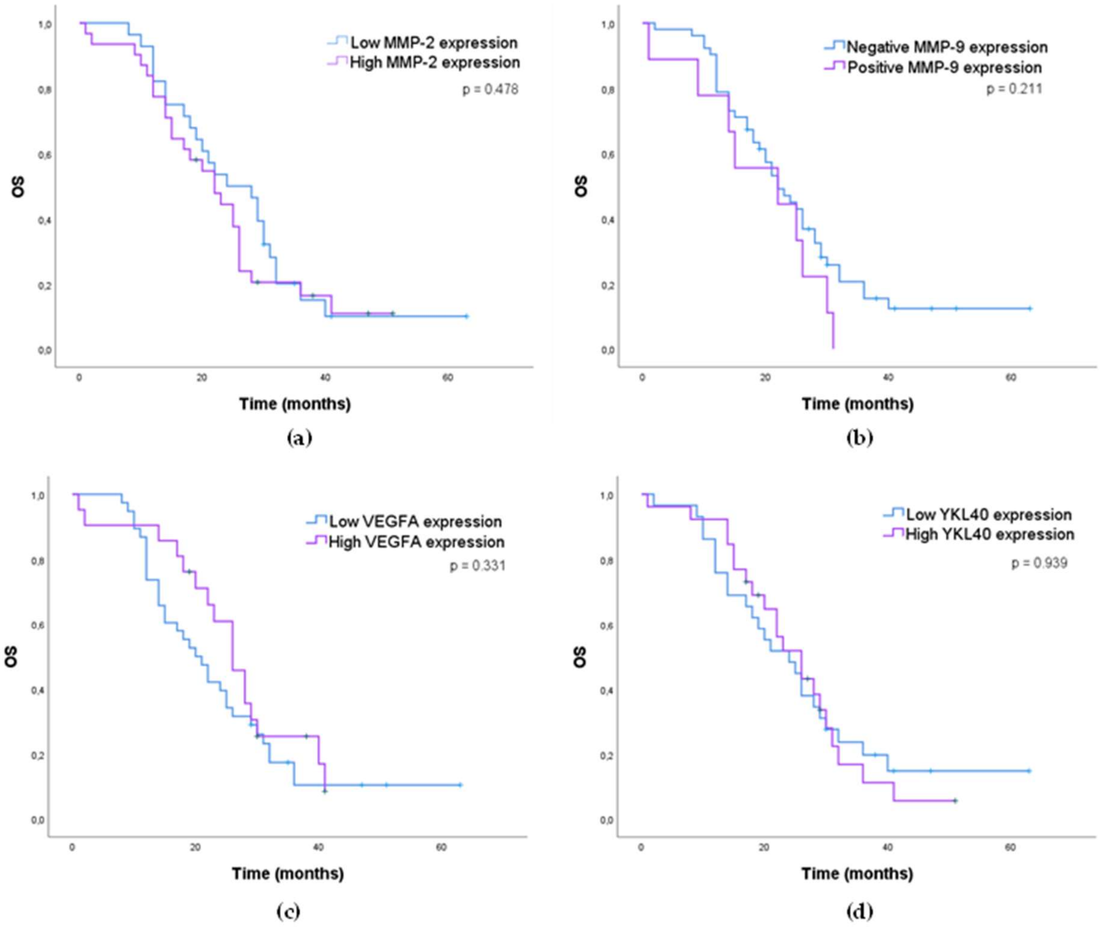


**Supplementary Figure S3.** Kaplan-Meier analysis of overall survival (OS - in months) according to the expression of MMP-2 **(a)**, MMP-9 **(b)**, VEGFA **(c)**, and YKL40 **(d)**.

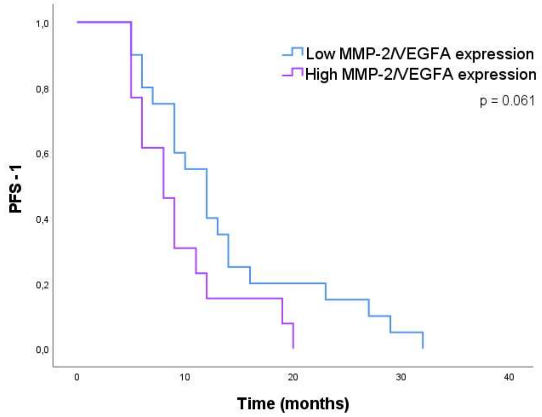


**Supplementary Figure S4.** Kaplan-Meier analysis of progression-free survival (in months) after temozolomide treatment (PFS-1) according to the concomitant expression of MMP-2 and VEGFA.

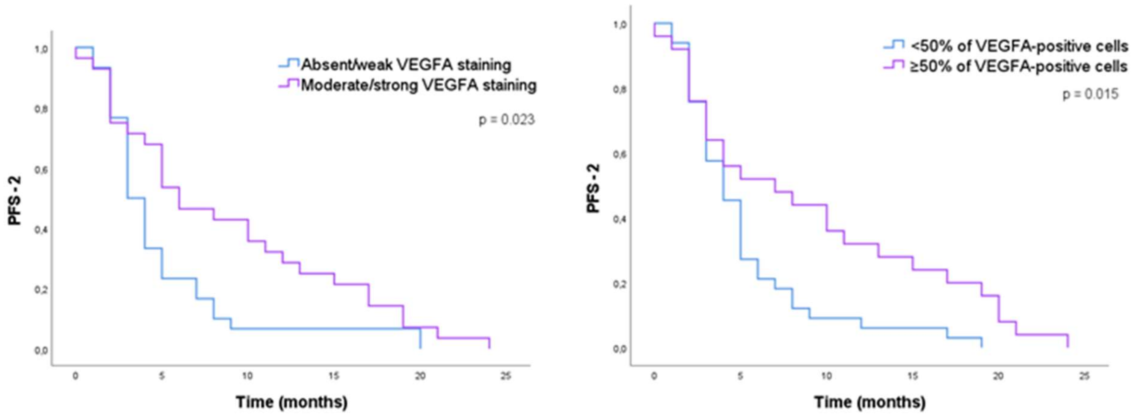


(a) (b)

**Supplementary Figure S5.** Kaplan-Meier analysis of progression-free survival (in months) after bevacizumab treatment (PFS-2) according to VEGFA staining intensity **(a)** and percentage of positive cells **(b)**.

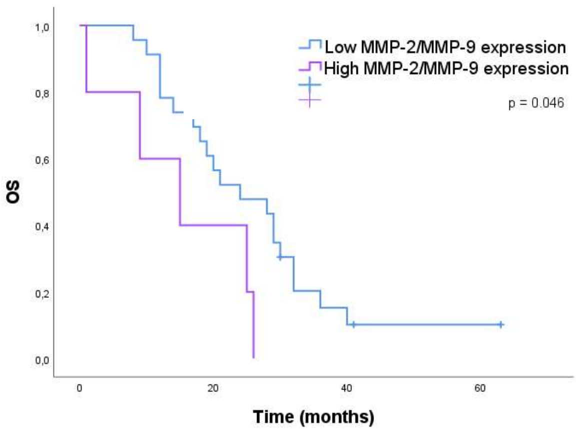


**Supplementary Figure S6.** Kaplan-Meier analysis of overall survival (OS - in months) according to the concomitant expression of MMP-2 and MMP-9.
